# Shared Sheath Rupture, an Uncommon Presentation of Acute Aortic Syndrome: A Case Report and Literature Review

**DOI:** 10.7759/cureus.115070

**Published:** 2026-08-24

**Authors:** Ethan Clarke, Mohammed Abubakr, Sarath Vennam, Andrew Edwards, Henry Sweeney

**Affiliations:** 1 Radiology, Royal Cornwall Hospitals National Health Service (NHS) Trust, Truro, GBR; 2 Cardiology, Royal Cornwall Hospitals National Health Service (NHS) Trust, Truro, GBR; 3 Interventional Radiology, Royal Cornwall Hospitals National Health Service (NHS) Trust, Truro, GBR; 4 Oncology, St George's Hospital, London, GBR

**Keywords:** acute aortic syndrome, ct angiography, penetrating aortic ulcer, radiology, shared sheath rupture

## Abstract

Shared sheath rupture is a very rare form of acute aortic syndrome (AAS) that requires urgent diagnosis and management. We report the case of an 84-year-old gentleman who presented with acute chest pain, initially suspected to be ischaemic in origin. Elevated troponin supported a working diagnosis of acute coronary syndrome (ACS) and treatment was initiated; however, the patient subsequently deteriorated. Computed tomography angiography (CTA) was requested with suspicion of AAS. It revealed a penetrating aortic ulcer with haematoma extending into the shared adventitial space between the aorta and pulmonary trunk, the so-called ‘shared sheath’. The patient was transferred for emergency surgical management. This case highlights a distinctly rare radiological finding, emphasising the importance of careful CTA interpretation, and illustrates the diagnostic challenges in differentiating AAS from ACS at presentation.

## Introduction

Acute aortic syndrome (AAS) encompasses a spectrum of life-threatening conditions characterised by disruption of the aortic wall. It is uncommon, with an estimated incidence of 4.8 per 100,000 [1]. The term was first introduced by Vilacosta et al. [2] to describe three main pathologies: aortic dissection (AD), intramural haematoma (IMH), and penetrating aortic ulcer (PAU). These are closely related pathologies, both in pathophysiology and clinical presentation, and may evolve from one to another. Patients with AAS typically describe sudden-onset central chest pain, often described as tearing, pulsatile, or burning, and the pain may migrate as the disease progresses, reflecting the extension of the underlying pathology, which may contribute to diagnostic uncertainty and challenge [3].

The incidence of each condition described within the AAS spectrum is different. AD is the most common, accounting for 70%-80% of reported cases [4]. IMH is the next most common (5%-25%) and PAU is the rarest (2%-7%) [4]. PAU was first described by Shennan in 1934 [5]. The underlying pathophysiology is atherosclerosis, with an ulcer that arises from the aortic wall and penetrates the elastic lamina [6]. The risk of rupture depends on the location of the ulcer [7]. In the setting of aortic rupture from an ascending aortic PAU, rupture can occur into an important space between the aorta and pulmonary trunk. The aorta and pulmonary trunk share an adventitial layer. When rupture from a penetrating ulcer occurs, blood can collect in this space as a haematoma. This is termed ‘shared sheath rupture’, a rare sequela of AAS, that has been sparsely described in the literature.

This case report describes a case of shared sheath rupture secondary to PAU, highlighting unusual and interesting radiological findings. It also emphasises the diagnostic challenges for the multiple specialities involved in assessing such a condition, most notably radiology. The review of literature addresses key concepts relating to AAS and aims to expand the current understanding of rare findings within AAS, specifically PAU and shared sheath rupture, with particular attention to the radiological evaluation of these findings.

## Case presentation

An 84-year-old male patient was brought to the emergency department by ambulance in the early hours of the morning with central chest pain. His medical history included hypertension, transient ischaemic attack, central retinal artery occlusion and branch retinal vein occlusion. He was otherwise functionally independent, and physically active, being noted to cycle long distances multiple times per week.

The patient described sudden-onset mild indigestion-like central chest pain of approximately 24 hours duration. He denied radiation or any exacerbating or relieving factors of the pain. The pain worsened with progressive malaise and three episodes of vomiting was reported later in the day. Initial physical examination was unremarkable, with vital signs within normal ranges, other than the patient’s blood pressure, which was initially stable but lower than his baseline.

**Figure 1:**
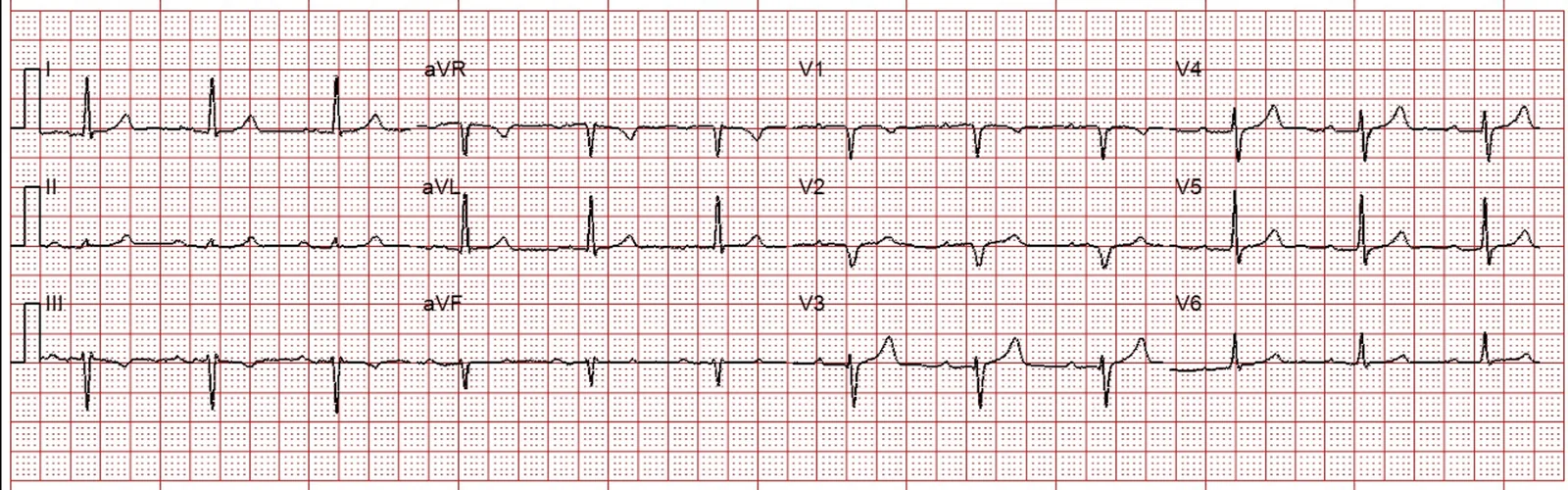
ECG of the patient showing sinus rhythm with first degree heart block.

Electrocardiogram (ECG) on admission demonstrated sinus rhythm with first degree heart block (Figure 1). Laboratory investigations revealed elevated Troponin I of 125ng/L, mildly raised white cell count of 11.2x10^9^/L, mild C-reactive protein (CRP) rise of 8 mg/L, along with moderate deterioration of kidney function (Table 1). Chest radiography showed cardiomegaly, tortuosity of the thoracic aorta and central pulmonary vascular prominence, with clear lung fields and pleural spaces. The patient was admitted under the cardiology team and treated initially as a case of acute coronary syndrome.

**Table 1:** Patient laboratory investigation results with reference ranges.

PARAMETERS	Patient values	Reference range
White cell count (WCC), x10^9^/L	11.2	3.7-9.5
C-Reactive protein (CRP), mg/L	8	0-5
Troponin I, ng/L	125	0-34
Estimated glomerular filtration rate (eGFR), mL/min	49	>90

**Figure 2:**
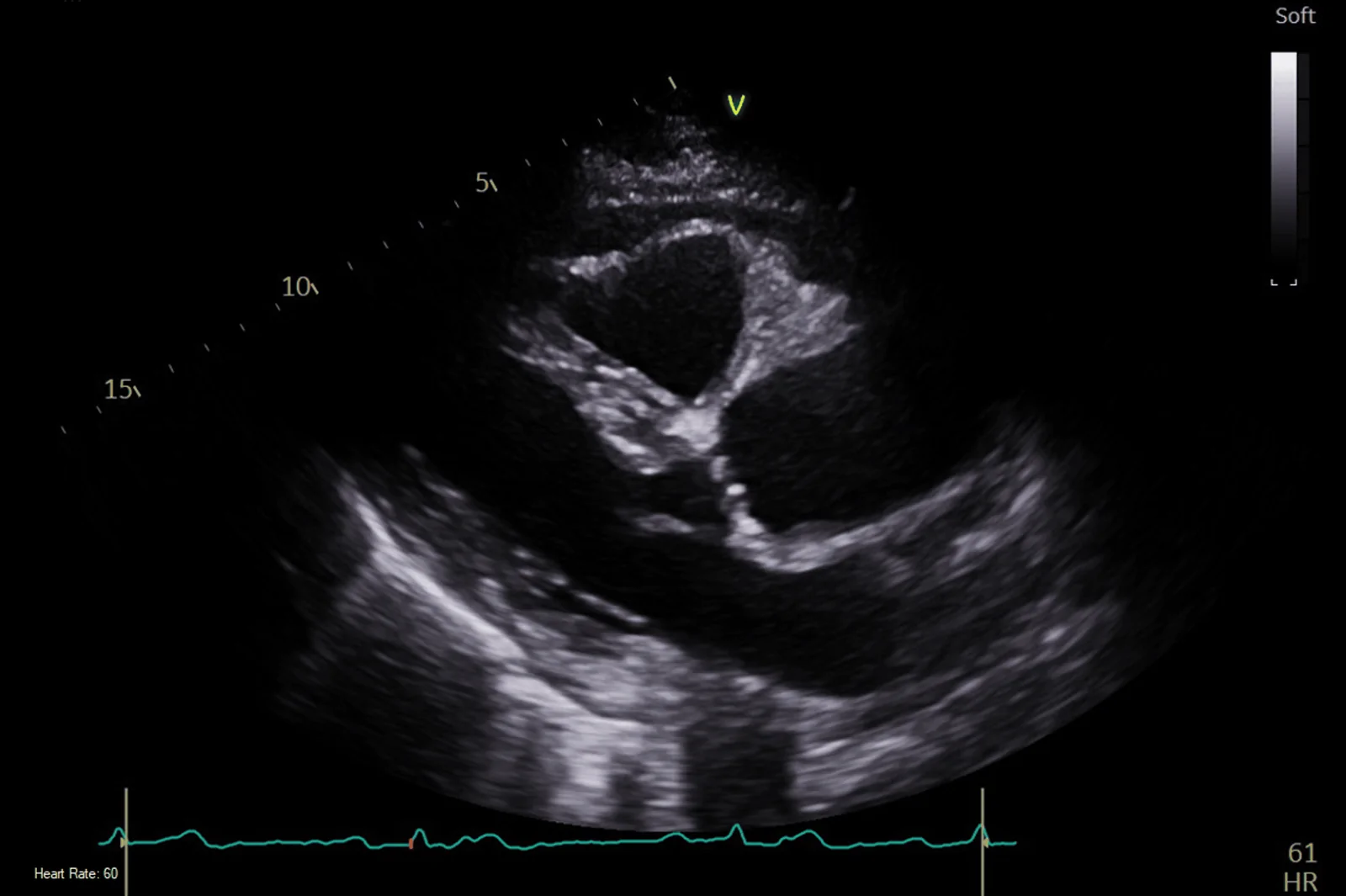
Transthoracic echocardiography (TTE) image showing pericardial effusion.

**Figure 3:**
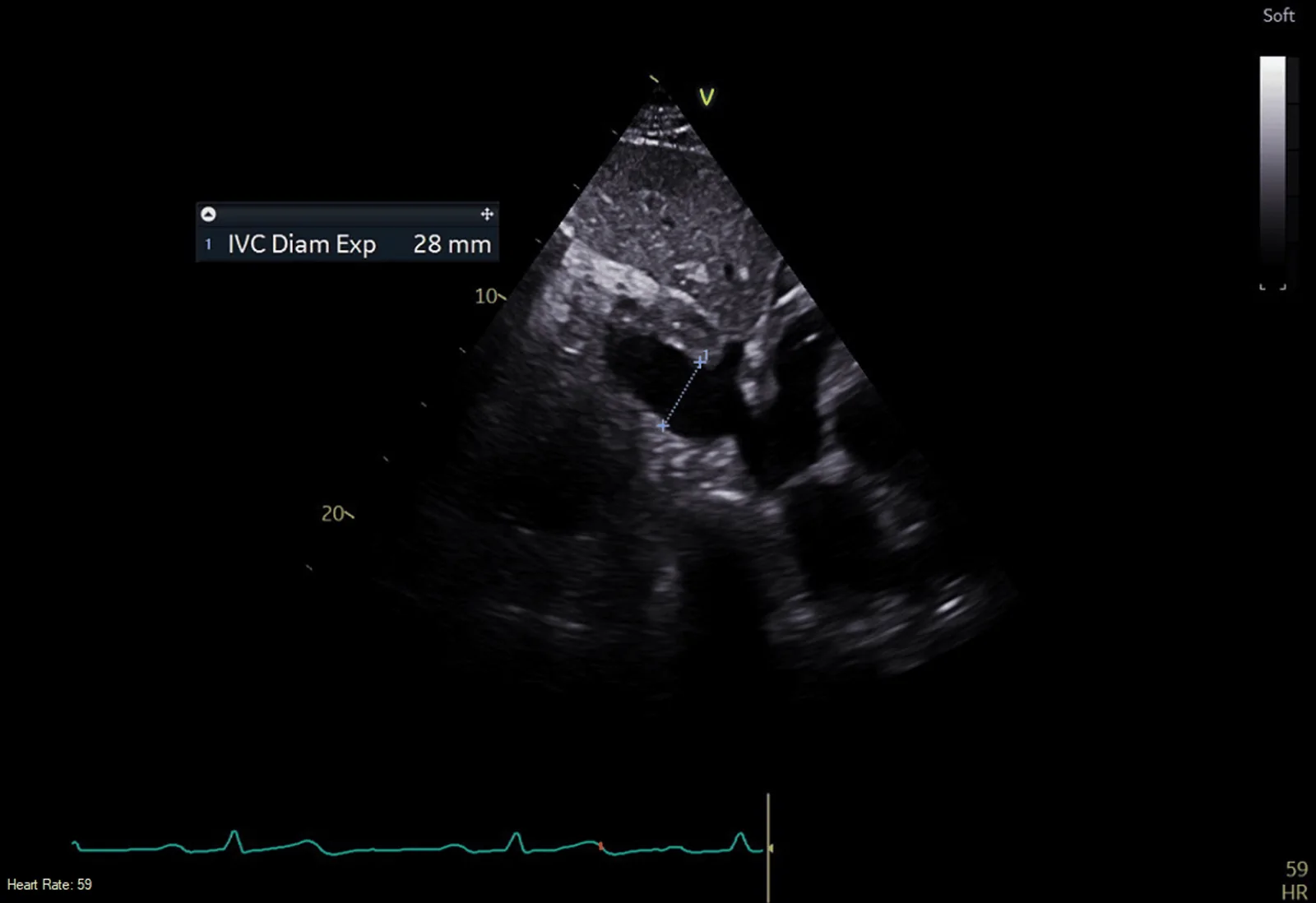
Transthoracic echocardiography (TTE) image showing plethoric inferior vena cava (IVC).

**Figure 4:**
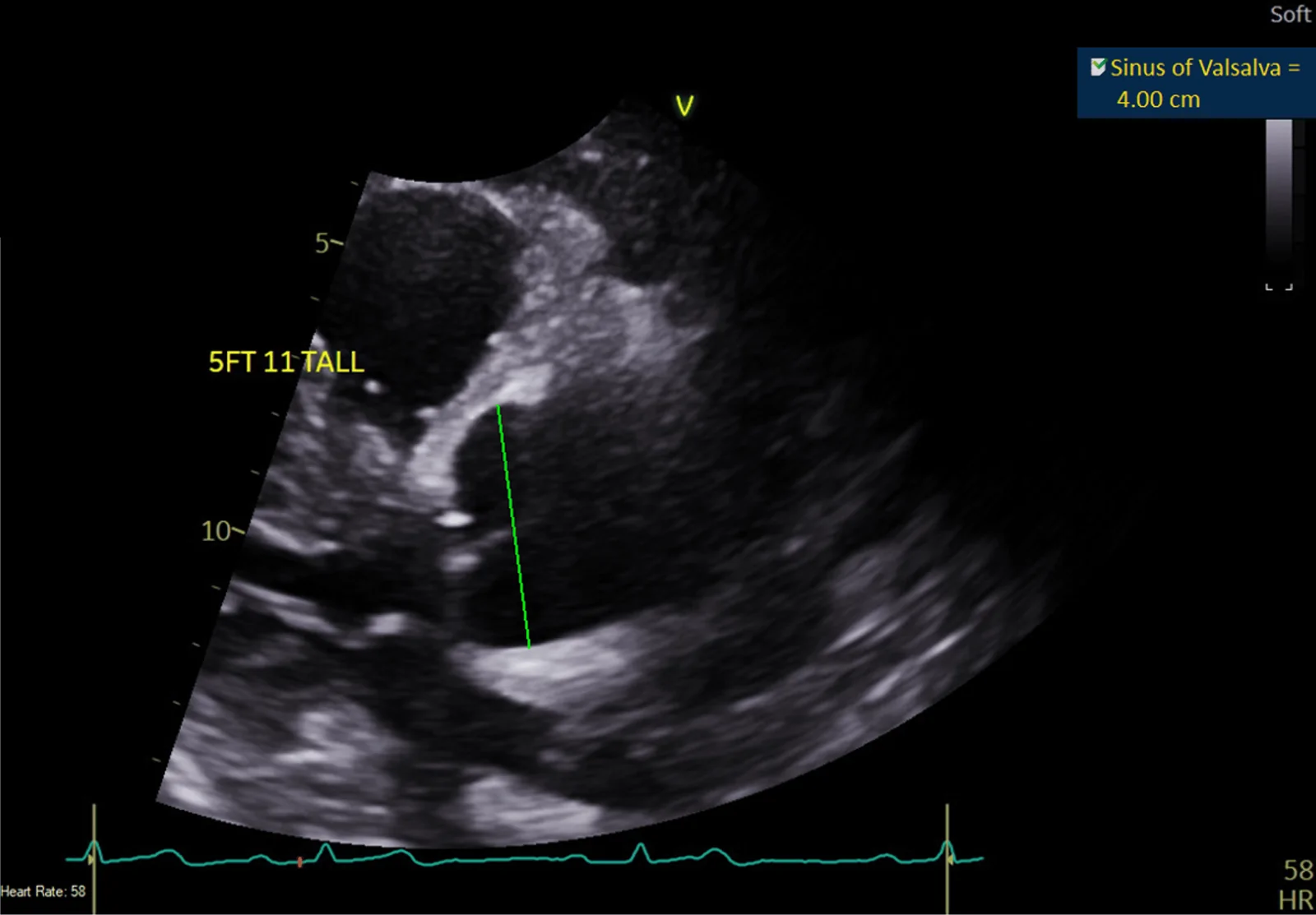
Transthoracic echocardiography (TTE) image showing dilated aortic root.

**Figure 5:**
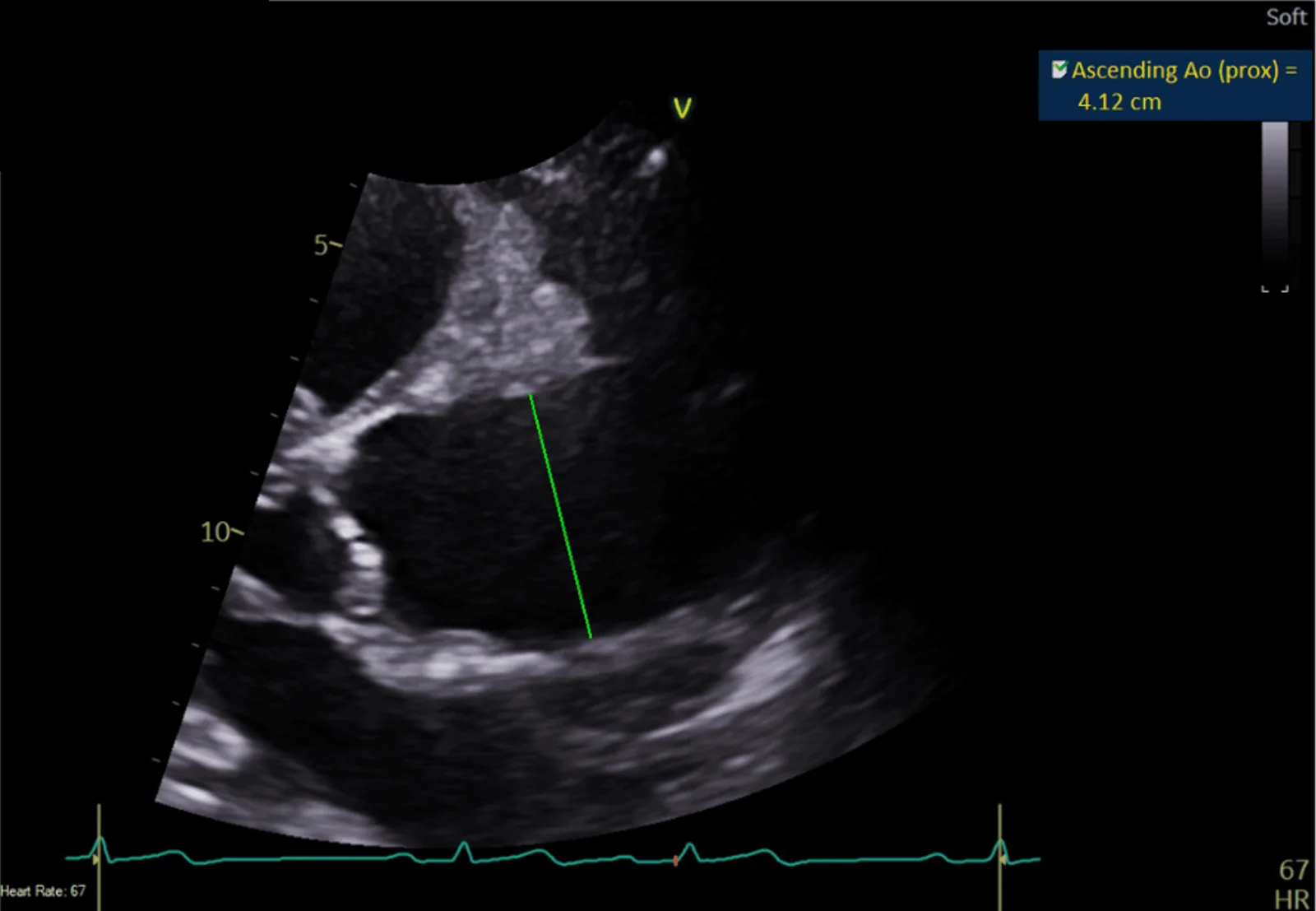
Transthoracic echocardiography (TTE) image showing dilated proximal ascending aorta.

Urgent transthoracic echocardiography (TTE) was requested by the cardiology team. This showed a global pericardial effusion (Figure 2), plethoric inferior vena cava (IVC) (Figure 3), and a dilated aortic root (Figure 4) and proximal ascending aorta (Figure 5). The patient’s ejection fraction was preserved.

**Figure 6:**
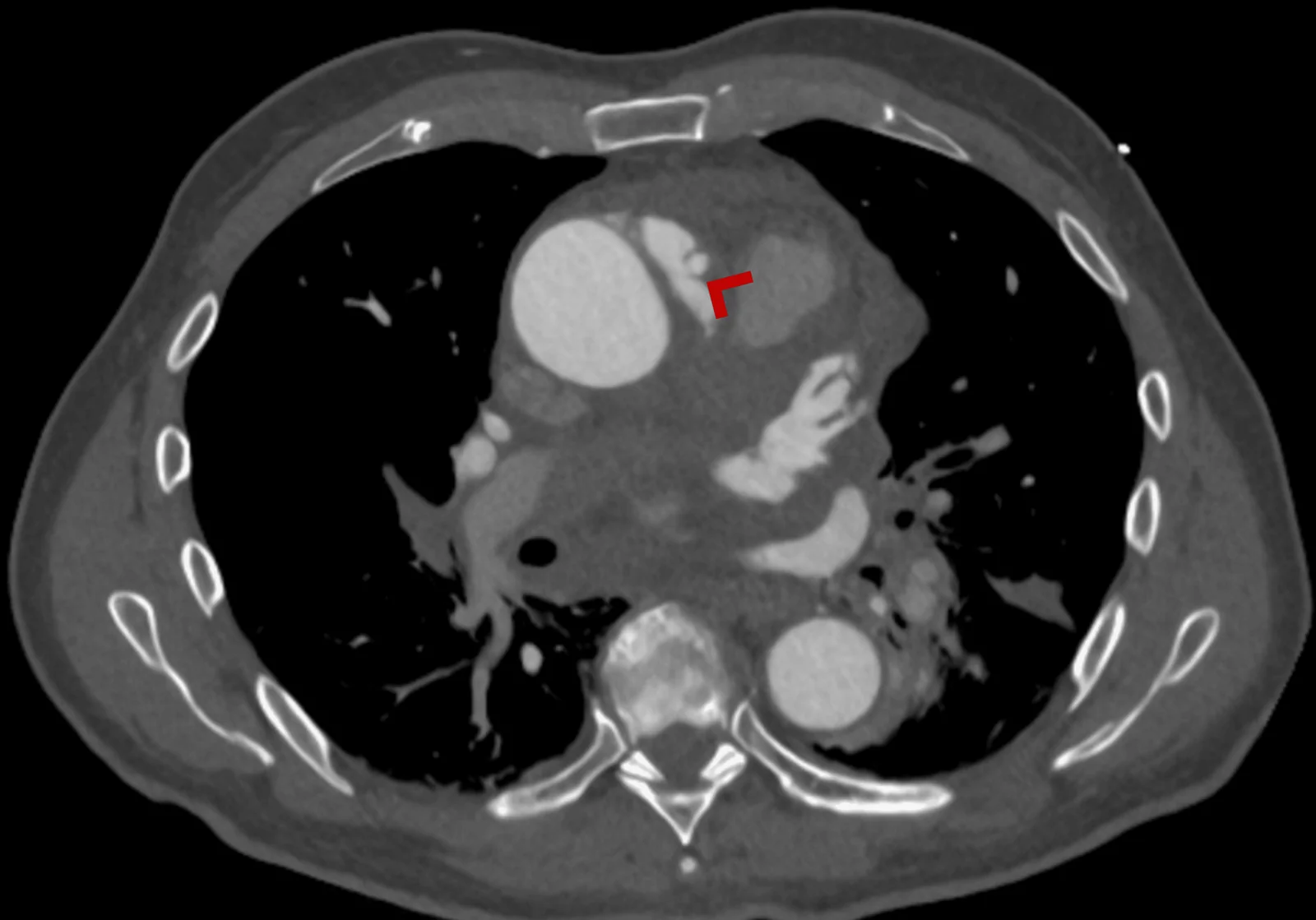
CT scan, axial arterial phase imaging. The red arrow indicates a focal outpouching of the ascending aorta, with surrounding haemopericardium; these features overall are in keeping with shared sheath aortic rupture.

**Figure 7:**
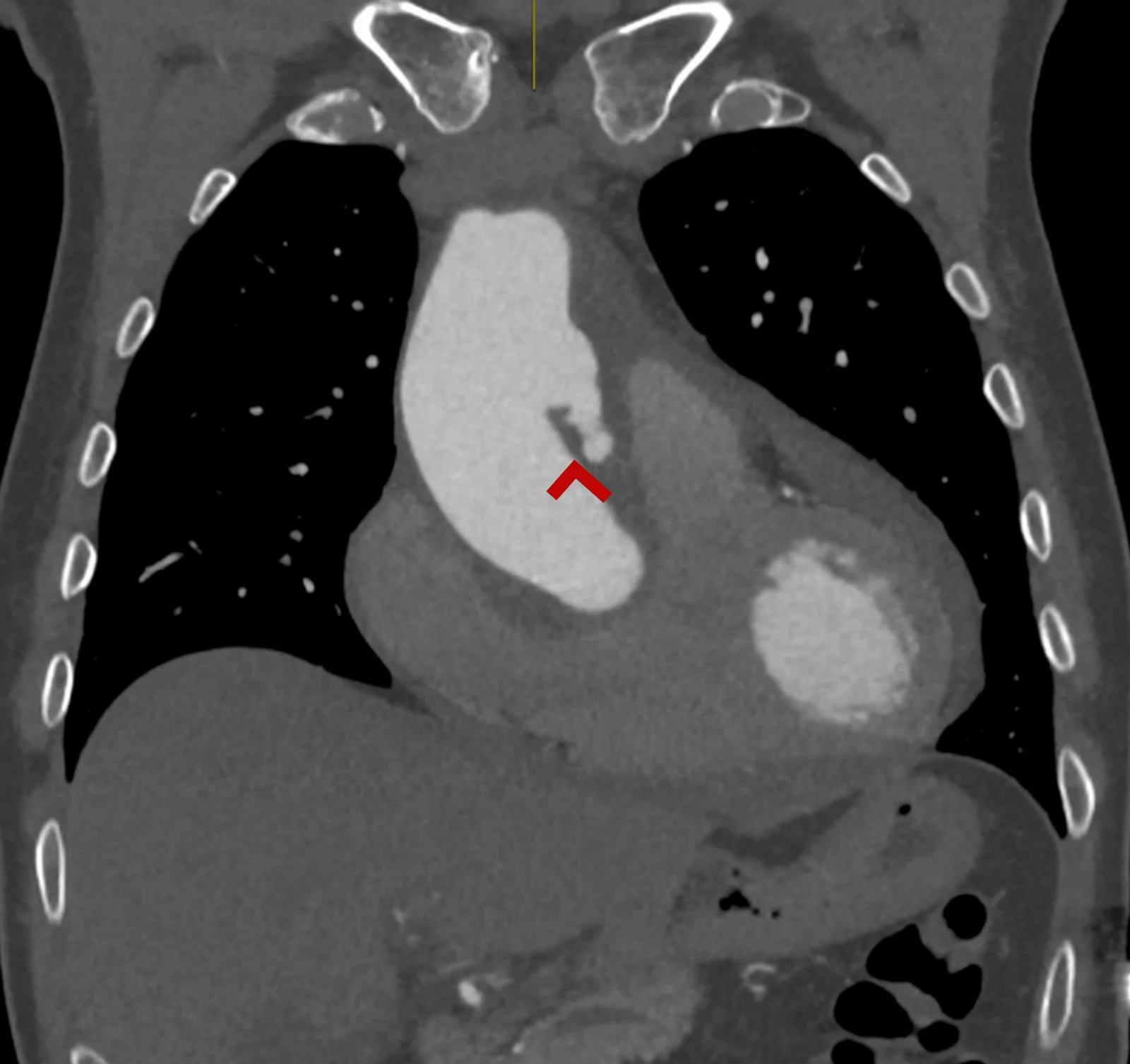
CT scan, coronal multiplanar reformat. The red arrow indicates outpouching of the ascending aorta.

**Figure 8:**
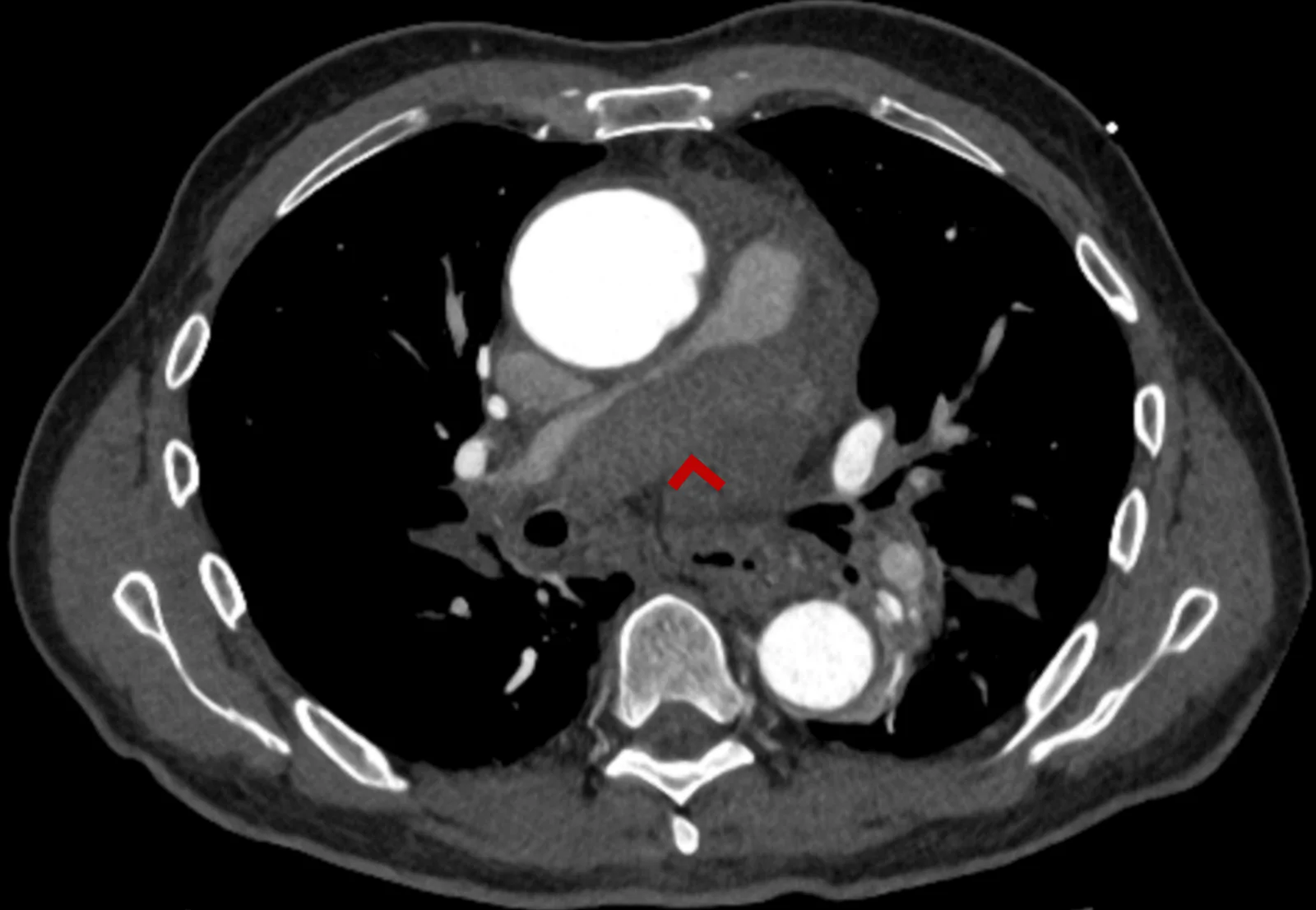
CT scan, axial arterial phase imaging. The red arrow indicates haematoma within the sheath of the great vessels partially effacing the pulmonary trunk.

**Figure 9:**
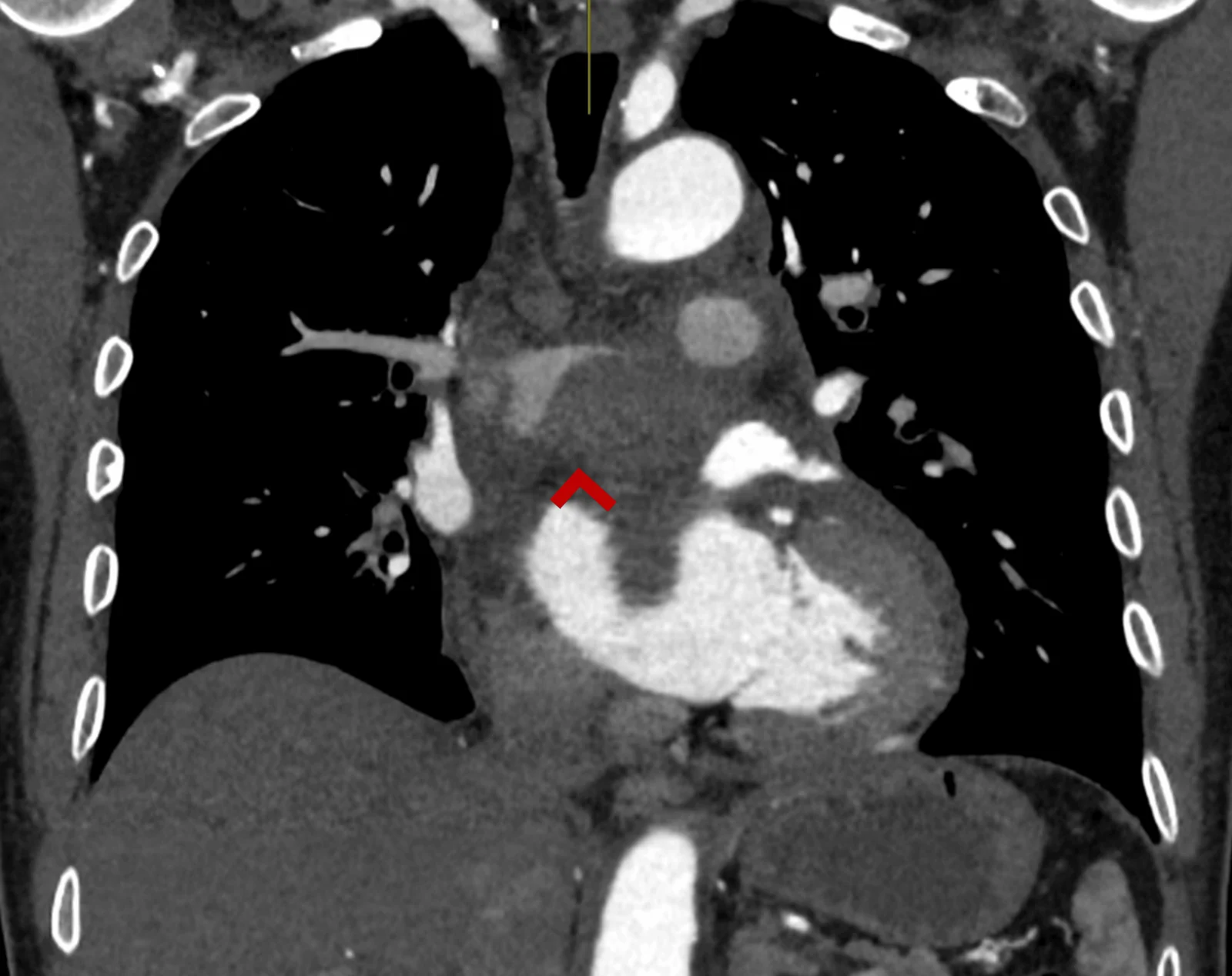
CT scan, coronal multiplanar reformat. The red arrow indicates haematoma within the sheath of the great vessels partially effacing the pulmonary trunk.

Subsequently, the patient developed acute hypotension with a new oxygen requirement. Planned urgent coronary angiography was deferred and urgent gated computed tomography (CT) of the thorax was performed. This revealed a penetrating ulcer in the proximal ascending aorta, demonstrated by a focal outpouching (Figures 6, 7) with associated haematoma, effacing the pulmonary trunk (Figures 8, 9), and a pericardial effusion, consistent with haemopericardium (Figure 6). This was classified as a Stanford type A acute aortic syndrome (AAS). The patient was transferred emergently to the regional cardiothoracic surgical centre for surgical management.

## Discussion

The AAS spectrum predominantly affects elderly patients, with higher incidence in males, and those with hypertension [8]. Additional risk factors include connective tissue disorders (e.g. Ehlers-Danlos or Marfan syndrome) [4], aortic valve disease, aneurysmal disease, and prior aortic instrumentation [1]. In this case, the patient’s primary risk factors were age, sex, and hypertension.

Chest pain is the most common presenting symptom, occurring in approximately 80% of cases [9]. The pain is classically described as tearing or ripping, but this is only present in 30%-40% of cases [10]. Pain may instead be burning or non-specific, as in this case, contributing to diagnostic ambiguity. Radiation of pain, although characteristic, is also inconsistently present [10].

Physical examination in AAS is often normal, as in this case; however, on occasion patients may present with findings such as a new aortic regurgitation murmur or new neurological deficit [1]. When looking at patient observations, haemodynamic status is key, with hypotension being a worrying sign, especially when considering aortic rupture. Other clinical examination features to consider include a pulse deficit or blood pressure differential [11]. The diagnostic accuracy of these latter two signs, however, is limited [12].

Biochemical markers are of limited specificity. Troponin elevation, as seen in this patient, can occur in AAS and may lead to misdiagnosis as acute coronary syndrome (ACS) [13]. D-dimer has demonstrated high sensitivity in AAS and may have a role in excluding the diagnosis, although it was not utilised in this case [14].

Imaging is central to the diagnosis of AAS. Plain chest radiography can be useful in excluding differentials and may demonstrate signs indicative of AAS but is not sensitive. These signs include mediastinal widening or tracheal shift; as in this case, chest X-ray may show tortuosity of the thoracic aorta alongside pulmonary vascular prominence and cardiomegaly.

CT angiography (CTA) is the gold standard for diagnosis [4] as demonstrated in this case, with a reported sensitivity and specificity approaching 100% [15]. CTA is commonly ECG-gated, which reduces motion artefact, resulting in improved imaging diagnostic accuracy compared to non-ECG-gated CTAs, which have a reported sensitivity of up to 93% [15], which results in diagnostic challenges. It is important to note, however, that scan gating can result in higher radiation exposure.

Biphasic or triphasic CT is often done when imaging patients with suspected AAS. The various phases each have important roles to play in elements such as evaluating presence and morphology of aortic pathology and assessing distal organ perfusion. The role of other imaging modalities (such as magnetic resonance (MR) angiogram and TTE) is limited. TTE may demonstrate complications related to AAS, such as aortic regurgitation [4] or pericardial effusion (haemopericardium) [16].

Cross-sectional imaging in general holds importance in the classification of AAS according to the Stanford system. Dissection (or another AAS) affecting the aorta proximal to the left subclavian artery is classified as Type A, whereas distal to the left subclavian artery is classified as type B [17]. This classification is important as Type A pathologies more often require surgical management. The Society for Vascular Surgery/Society of Thoracic Surgeons (SVS/STS) also outlines an alternative system with zones 0-12 [17]. Cross-sectional imaging overall is key in the diagnosis of AAS; CTA in this case demonstrated Stanford type A AAS, with the presence of a PAU and haemopericardium.

PAU is the rarest of the three conditions that make up the AAS spectrum [4] and depending on the location can pose a distinct risk of aortic rupture. The acronym PAU is often used for penetrating aortic ulcer, but can be used for penetrating atherosclerotic ulcer, which are one and the same, reflecting the underlying pathophysiology of the condition. A PAU involves an ulcerating lesion that penetrates through the tunica intima of the aortic wall. This then progresses into the tunica media, and blood can then collect as an intramural haematoma. This is different from the intramural haematoma described as one of the types of AAS, as this type has no disturbance of the tunica intima; in this case, spontaneous rupture of the vasa vasorum produces isolated haematoma within the tunica media.

Common CT findings for a PAU include a protrusion of the aortic wall outwards, or a similarly shaped protrusion into the aortic wall without the outer wall protruding out. The shape of the protrusion in either case is sometimes described as looking like a mushroom [18]. Associated intramural haematoma is common [19]. Given the pathophysiology of the condition being atherosclerosis, appearance of atherosclerosis in other areas such as the coronary vessels can be a useful finding to raise the possibility of PAU. A PAU can be found anywhere in the aorta, but occurrence in the descending aorta is most common, accounting for 60%-70% of cases [19]; arch and ascending aorta occurrence is much rarer, so in this way location of pathology can inform imaging interpretation.

A PAU can progress in several ways. This can be to form aneurysms, cause dissection, or progress to aortic rupture. In some cases, however, they can spontaneously resolve, or sometimes do not resolve but remain stable without progression; this is common for PAUs with a small neck width, or those found in the descending aorta, which can be managed conservatively [20]. A PAU of the ascending aorta, as was the case for this patient and thus classifying their AAS as Stanford A, has a very high risk of rupture [7] and so necessitates rapid surgical management. In the setting of aortic rupture from an ascending aortic PAU, rupture can occur into an important space between the aorta and pulmonary trunk, as it did for this patient.

The aorta and pulmonary trunk share an adventitial layer. When rupture from a penetrating ulcer occurs, blood can collect in this space as a haematoma. This is termed ‘shared sheath rupture’. This haematoma can extend along this shared space and compress associated vessels. CT angiography in this patient showed a penetrating ulcer in the ascending aorta with haematoma, and related compression of the right pulmonary artery. From a radiological standpoint, this represents a dilemma as this compression of pulmonary vasculature on CT angiography can mimic a pulmonary embolism. If the scan is then subsequently reported as a pulmonary embolism, this has huge implications for the patient who may then go on to be treated with anticoagulant or thrombolytic therapy for suspected thrombotic disease, when in fact they are haemorrhaging; this could have catastrophic consequences for the patient. Radiologists need to be assiduous when interpreting scans such as this to differentiate between intraluminal pathology such as pulmonary embolism, and extraluminal pathology such as compressing haematoma, to guide patient management effectively.

## Conclusions

AAS is a complex, relatively rare and potentially fatal condition requiring rapid diagnosis and intervention to maximise the chances of positive outcome. Shared sheath rupture is an exceptionally rare complication of PAU and represents an important radiological entity. Its imaging features may mimic other pathologies, particularly pulmonary embolism, with significant implications for management.

This case illustrates the diagnostic challenges in distinguishing AAS from ACS, particularly in the presence of elevated troponin and non-specific symptoms. The radiology team also face challenges; the imaging of shared sheath rupture can be difficult to interpret and can present diagnostic pitfalls which could lead to serious adverse outcomes for the patient. This patient was acutely unwell and went onto have urgent surgical management of their condition, the speed of which was greatly impacted by efficient radiological diagnosis. Increased awareness of this phenomenon, alongside careful interpretation of CTA, is essential to avoid misdiagnosis and ensure appropriate treatment. Early recognition and timely surgical intervention remain critical to improving patient outcomes.
